# Diaqua­bis­[1,2-bis­(pyridin-4-yl)ethene]­bis­[2-(4-carboxy­phen­yl)acetato]­cobalt(II)

**DOI:** 10.1107/S1600536811024755

**Published:** 2011-06-30

**Authors:** Wei-Hua Yu, Jian-Lan Liu, Xiao-Ming Ren

**Affiliations:** a College of Science, Nanjing University of Technology, Nanjing 210009, People’s Republic of China

## Abstract

The asymmetric unit of the title compound, [Co(C_9_H_7_O_4_)_2_(C_12_H_10_N_2_)_2_(H_2_O)_2_], consists of one Co^2+^ ion, one mono-deprotonated 2-(4-carboxyl­atophen­yl)acetate carboxylic acid, one 1,2-bis­(pyridin-4-yl)ethane mol­ecule and one water mol­ecule. The Co^II^ atom is situated on a crystallographic center of inversion and is octa­hedrally coordinated by two O atoms from two anions, two N atoms of two 1,2-bis­(pyridin-4-yl)ethane mol­ecules and two O atoms from two water mol­ecules. A three-dimensional network is established by inter­molecular O—H⋯O and O—H⋯N hydrogen bonds.

## Related literature

For general background to the design of metal-organic supra­molecular solids with potential functionality, see: Moulton & Zaworotko (2001 ▶); Janiak (2003 ▶). For weak non-covalent inter­actions in supra­molecular solids, see: Hosseini (2005 ▶); Nishio (2004 ▶). For metal-organic supra­molecular frameworks based on organic connectors containing pyridyl and/or carboxyl­ate groups, see: Brammer (2004 ▶).
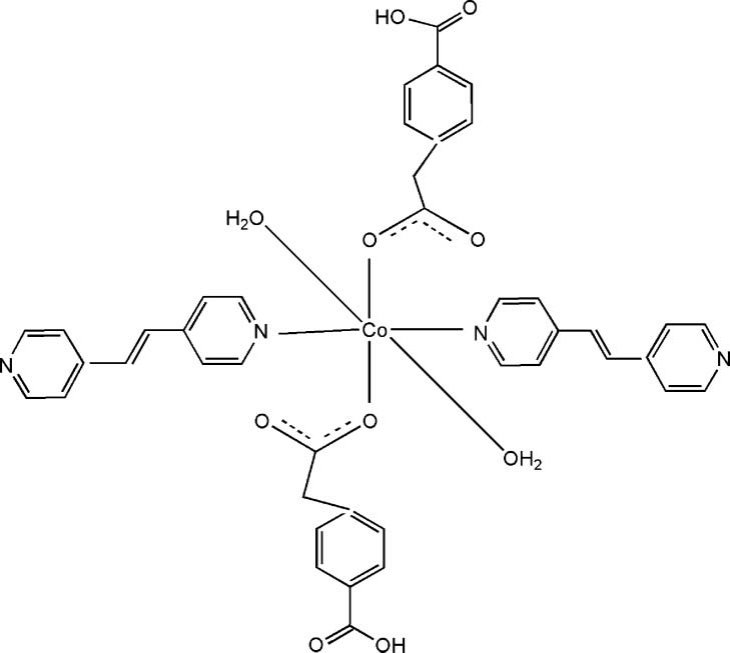


## Experimental

### 

#### Crystal data

[Co(C_9_H_7_O_4_)_2_(C_12_H_10_N_2_)_2_(H_2_O)_2_]
*M* *_r_* = 817.69
Monoclinic, 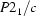

*a* = 21.349 (5) Å
*b* = 5.6522 (12) Å
*c* = 15.659 (4) Å
β = 98.999 (4)°
*V* = 1866.3 (7) Å^3^
*Z* = 2
Mo *K*α radiation
μ = 0.53 mm^−1^
*T* = 293 K
0.40 × 0.30 × 0.10 mm

#### Data collection

Bruker SMART CCD area-detector diffractometer
Absorption correction: multi-scan (*SADABS*; Bruker, 2000 ▶) *T*_min_ = 0.850, *T*_max_ = 0.874
9499 measured reflections
3635 independent reflections
2640 reflections with *I* > 2σ(*I*)
*R*_int_ = 0.041

#### Refinement

*R*[*F* ^2^ > 2σ(*F* ^2^)] = 0.055
*wR*(*F* ^2^) = 0.105
*S* = 1.06
3635 reflections
268 parameters
H atoms treated by a mixture of independent and constrained refinement
Δρ_max_ = 0.71 e Å^−3^
Δρ_min_ = −0.36 e Å^−3^

### 

Data collection: *SMART* (Bruker, 2000 ▶); cell refinement: *SAINT* (Bruker, 2000 ▶); data reduction: *SAINT*; program(s) used to solve structure: *SHELXS97* (Sheldrick, 2008 ▶); program(s) used to refine structure: *SHELXL97* (Sheldrick, 2008 ▶); molecular graphics: *SHELXTL* (Sheldrick, 2008 ▶); software used to prepare material for publication: *SHELXTL*.

## Supplementary Material

Crystal structure: contains datablock(s) global, I. DOI: 10.1107/S1600536811024755/im2300sup1.cif

Structure factors: contains datablock(s) I. DOI: 10.1107/S1600536811024755/im2300Isup2.hkl

Additional supplementary materials:  crystallographic information; 3D view; checkCIF report

## Figures and Tables

**Table 1:** Hydrogen-bond geometry (Å, °)

*D*—H⋯*A*	*D*—H	H⋯*A*	*D*⋯*A*	*D*—H⋯*A*
O5—H5*C*⋯O1^i^	0.73 (4)	2.13 (4)	2.822 (3)	158 (4)
O5—H5*D*⋯O2^ii^	0.98 (4)	1.74 (4)	2.610 (3)	145 (3)
O3—H3⋯N2^iii^	0.82	1.85	2.667 (3)	173

Symmetry codes: (i) 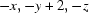; (ii) 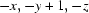; (iii) 
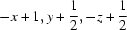
.

**Fig. 1.**
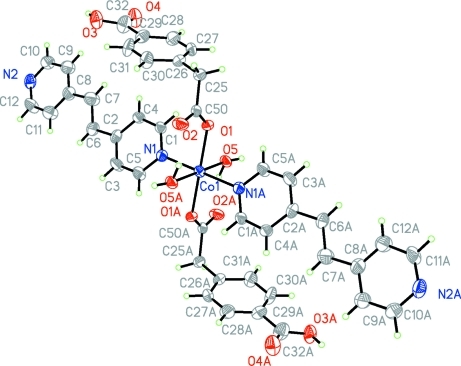
Molecular structure of (I), showing displacement ellipsoids at the 30% probability level.

**Fig. 2.**
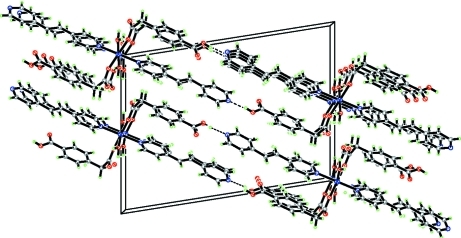
One-dimensional chain structure of (I).

## supplementary crystallographic information

### Comment

During the past decade, the design of new metal-organic supramolecular solids has attracted ever-increasing focus in the fields of coordination chemistry and crystal engineering, for the sake of developing desired crystalline materials with potential functionality (Moulton & Zaworotko, 2001; Janiak *et al.*, 2003). Furthermore, it has been realised that weak noncovalent interactions such as hydrogen bonds, aromatic stacking, and van der Waals forces (Hosseini, 2005; Nishio, 2004) are crucial in the direction of such crystalline architectures. Hitherto, a variety of organic connectors containing pyridyl and/or carboxylate groups (Brammer, 2004) have been widely used to construct metal-organic supramolecular frameworks. Herein we report the crystal structure of the title compound (1).

The molecular structure of (1) is illustrated in Fig. 1. Compound (1) crystallizes in the monoclinic space group P2_1_/c. The structure of (1) is a single molecule, in which the Co^2+^ center is situated on a crystallographic center of inversion. The coordination sphere of cobalt is a slightly distorted octahedron and consistes of by two O atoms from two mono-deprotonated (4-carboxyphenyl)acetate groups, two N atoms of two 1,2-di(pyridin-4-yl)ethane molecules and two O atoms from two water molecules. As shown in Fig. 2, a one-dimensional chain is formed by O–H···N hydrogen  bonds. In addition, these one-dimensional chains are linked together by additional O—H···O hydrogen bonds between water molecules and the cobalt bound carboxylate group generating a three-dimensional framework.

### Experimental

Cobalt chloride hexahydrate (1 mmol), 1,2-di(pyridin-4-yl)ethane (1 mmol) and (4-carboxyphenyl)acetic acid (1 mmol) in water (8 ml) were placed in a Teflon-lined stainless-steel Parr bomb that was heated to 433 K for 48 h. Red plate crystals were collected after the bomb was subsequently allowed to cool to room temperature (yield: 38%).

### Refinement

C-bound H atoms were placed geometrically (C—H = 0.93, and 0.98 Å) and refined as riding atoms, with *U*_iso_(H) = 1.2*U*_eq_(C). O-bound H atoms were located in difference Fourier maps and refined as riding in their as-found relative positions (O—H =0.96 Å) with *U*_iso_(H) = 1.5*U*_eq_(C).

### Crystal data

**Table:**

[Co(C_9_H_7_O_4_)_2_(C_12_H_10_N_2_)_2_(H_2_O)_2_]	*F*(000) = 850
*M**_r_* = 817.69	*D*_x_ = 1.455 Mg m^−^^3^
Monoclinic, *P*2_1_/*c*	Mo *K*α radiation, λ = 0.71073 Å
Hall symbol: -P 2ybc	Cell parameters from 870 reflections
*a* = 21.349 (5) Å	θ = 2.6–22.1°
*b* = 5.6522 (12) Å	µ = 0.53 mm^−^^1^
*c* = 15.659 (4) Å	*T* = 293 K
β = 98.999 (4)°	Plate, red
*V* = 1866.3 (7) Å^3^	0.40 × 0.30 × 0.10 mm
*Z* = 2	

### Data collection

**Table:**

Bruker SMART CCD area-detector diffractometer	3635 independent reflections
Radiation source: fine-focus sealed tube	2640 reflections with *I* > 2σ(*I*)
graphite	*R*_int_ = 0.041
φ and ω scans	θ_max_ = 26.0°, θ_min_ = 1.9°
Absorption correction: multi-scan (*SADABS*; Bruker, 2000)	*h* = −26→23
*T*_min_ = 0.850, *T*_max_ = 0.874	*k* = −6→6
9499 measured reflections	*l* = −19→17

### Refinement

**Table:**

Refinement on *F*^2^	Primary atom site location: structure-invariant direct methods
Least-squares matrix: full	Secondary atom site location: difference Fourier map
*R*[*F*^2^ > 2σ(*F*^2^)] = 0.055	Hydrogen site location: inferred from neighbouring sites
*wR*(*F*^2^) = 0.105	H atoms treated by a mixture of independent and constrained refinement
*S* = 1.06	*w* = 1/[σ^2^(*F*_o_^2^) + (0.040*P*)^2^ + 0.440*P*] where *P* = (*F*_o_^2^ + 2*F*_c_^2^)/3
3635 reflections	(Δ/σ)_max_ < 0.001
268 parameters	Δρ_max_ = 0.71 e Å^−^^3^
0 restraints	Δρ_min_ = −0.36 e Å^−^^3^

### Special details

**Table:**

Geometry. All e.s.d.'s (except the e.s.d. in the dihedral angle between two l.s. planes) are estimated using the full covariance matrix. The cell e.s.d.'s are taken into account individually in the estimation of e.s.d.'s in distances, angles and torsion angles; correlations between e.s.d.'s in cell parameters are only used when they are defined by crystal symmetry. An approximate (isotropic) treatment of cell e.s.d.'s is used for estimating e.s.d.'s involving l.s. planes.
Refinement. Refinement of *F*^2^ against ALL reflections. The weighted *R*-factor *wR* and goodness of fit *S* are based on *F*^2^, conventional *R*-factors *R* are based on *F*, with *F* set to zero for negative *F*^2^. The threshold expression of *F*^2^ > σ(*F*^2^) is used only for calculating *R*-factors(gt) *etc*. and is not relevant to the choice of reflections for refinement. *R*-factors based on *F*^2^ are statistically about twice as large as those based on *F*, and *R*- factors based on ALL data will be even larger.

### Fractional atomic coordinates and isotropic or equivalent isotropic displacement parameters (Å^2^)

**Table:**

	*x*	*y*	*z*	*U*_iso_*/*U*_eq_	
C1	0.13939 (13)	0.6024 (5)	0.07330 (17)	0.0285 (6)	
H1	0.1308	0.7388	0.0404	0.034*	
C2	0.21300 (13)	0.3771 (6)	0.16945 (19)	0.0324 (7)	
C3	0.16536 (14)	0.2132 (6)	0.1675 (2)	0.0418 (8)	
H3A	0.1727	0.0750	0.1997	0.050*	
C4	0.19847 (13)	0.5792 (5)	0.11978 (18)	0.0305 (7)	
H4	0.2287	0.6969	0.1183	0.037*	
C5	0.10794 (14)	0.2501 (5)	0.11927 (19)	0.0360 (7)	
H5A	0.0771	0.1338	0.1194	0.043*	
C6	0.27478 (13)	0.3325 (6)	0.2271 (2)	0.0446 (8)	
H6	0.2805	0.2017	0.2635	0.053*	
C7	0.32607 (15)	0.5046 (7)	0.2236 (2)	0.0542 (9)	
H7	0.3213	0.6348	0.1868	0.065*	
C8	0.38736 (14)	0.4545 (6)	0.2844 (2)	0.0435 (8)	
C9	0.43624 (15)	0.6125 (7)	0.2885 (2)	0.0480 (9)	
H9	0.4317	0.7491	0.2549	0.058*	
C10	0.49294 (15)	0.5670 (6)	0.3433 (2)	0.0418 (8)	
H10	0.5263	0.6730	0.3439	0.050*	
C11	0.45391 (15)	0.2330 (6)	0.3909 (2)	0.0467 (9)	
H11	0.4588	0.1017	0.4271	0.056*	
C12	0.39688 (15)	0.2605 (6)	0.3370 (2)	0.0476 (9)	
H12	0.3651	0.1477	0.3363	0.057*	
C25	0.09958 (12)	0.8462 (5)	−0.19283 (18)	0.0311 (6)	
H25A	0.1055	0.7968	−0.2504	0.037*	
H25B	0.0775	0.9968	−0.1978	0.037*	
C26	0.16362 (13)	0.8760 (5)	−0.13681 (18)	0.0290 (6)	
C27	0.17905 (14)	1.0732 (5)	−0.0867 (2)	0.0333 (7)	
H27	0.1497	1.1953	−0.0881	0.040*	
C28	0.23634 (15)	1.0942 (6)	−0.0351 (2)	0.0422 (8)	
H28	0.2446	1.2272	−0.0002	0.051*	
C29	0.28176 (14)	0.9246 (6)	−0.0335 (2)	0.0387 (7)	
C30	0.26777 (14)	0.7226 (6)	−0.0820 (2)	0.0447 (8)	
H30	0.2978	0.6031	−0.0811	0.054*	
C31	0.20875 (14)	0.6992 (6)	−0.1321 (2)	0.0408 (8)	
H31	0.1992	0.5607	−0.1635	0.049*	
C32	0.34424 (16)	0.9532 (6)	0.0221 (2)	0.0494 (9)	
C50	0.05974 (12)	0.6632 (5)	−0.15437 (17)	0.0237 (6)	
Co1	0.0000	0.5000	0.0000	0.02453 (15)	
H5C	−0.0226 (18)	0.907 (7)	0.078 (3)	0.057 (13)*	
H5D	−0.0072 (15)	0.719 (6)	0.134 (2)	0.049 (10)*	
N1	0.09278 (10)	0.4435 (4)	0.07147 (14)	0.0278 (5)	
N2	0.50139 (12)	0.3794 (5)	0.39444 (17)	0.0417 (6)	
O1	0.03952 (8)	0.7233 (3)	−0.08597 (11)	0.0252 (4)	
O2	0.05249 (10)	0.4649 (4)	−0.18973 (14)	0.0392 (5)	
O3	0.38617 (10)	0.7984 (4)	0.00934 (15)	0.0448 (6)	
H3	0.4191	0.8232	0.0426	0.067*	
O4	0.35646 (11)	1.1161 (4)	0.07192 (15)	0.0506 (6)	
O5	−0.00449 (10)	0.7965 (4)	0.07875 (14)	0.0308 (5)	

### Atomic displacement parameters (Å^2^)

**Table:**

	*U*^11^	*U*^22^	*U*^33^	*U*^12^	*U*^13^	*U*^23^
C1	0.0255 (14)	0.0263 (14)	0.0317 (15)	−0.0001 (11)	−0.0015 (11)	0.0051 (12)
C2	0.0228 (15)	0.0414 (18)	0.0314 (15)	0.0045 (12)	−0.0004 (12)	−0.0013 (13)
C3	0.0411 (17)	0.0279 (17)	0.052 (2)	−0.0034 (14)	−0.0078 (14)	0.0124 (15)
C4	0.0254 (14)	0.0280 (16)	0.0360 (16)	−0.0053 (11)	−0.0017 (12)	0.0017 (12)
C5	0.0329 (15)	0.0323 (17)	0.0372 (17)	−0.0017 (13)	−0.0112 (12)	0.0083 (13)
C6	0.0238 (16)	0.050 (2)	0.056 (2)	0.0032 (14)	−0.0049 (14)	0.0141 (17)
C7	0.0407 (18)	0.056 (2)	0.061 (2)	−0.0067 (18)	−0.0091 (15)	0.023 (2)
C8	0.0303 (16)	0.043 (2)	0.055 (2)	−0.0009 (14)	−0.0020 (13)	0.0019 (15)
C9	0.0383 (19)	0.050 (2)	0.053 (2)	−0.0036 (15)	−0.0021 (15)	0.0131 (17)
C10	0.0357 (17)	0.042 (2)	0.0446 (19)	−0.0083 (14)	−0.0035 (14)	−0.0042 (14)
C11	0.0469 (19)	0.0385 (19)	0.0453 (19)	−0.0024 (15)	−0.0222 (15)	0.0135 (15)
C12	0.0373 (18)	0.051 (2)	0.048 (2)	−0.0097 (16)	−0.0121 (15)	0.0094 (17)
C25	0.0254 (14)	0.0341 (17)	0.0340 (15)	−0.0005 (12)	0.0056 (12)	0.0059 (13)
C26	0.0241 (14)	0.0337 (16)	0.0301 (15)	−0.0018 (12)	0.0069 (11)	−0.0003 (12)
C27	0.0326 (16)	0.0210 (16)	0.0469 (18)	0.0048 (11)	0.0086 (13)	0.0006 (12)
C28	0.0408 (19)	0.0344 (18)	0.054 (2)	−0.0039 (14)	0.0135 (15)	−0.0099 (15)
C29	0.0296 (15)	0.044 (2)	0.0424 (17)	−0.0057 (13)	0.0056 (13)	−0.0033 (14)
C30	0.0256 (15)	0.052 (2)	0.054 (2)	0.0122 (15)	−0.0021 (13)	−0.0096 (17)
C31	0.0404 (17)	0.0326 (17)	0.0480 (19)	0.0022 (14)	0.0024 (14)	−0.0182 (14)
C32	0.0381 (18)	0.053 (2)	0.054 (2)	−0.0008 (16)	−0.0024 (15)	−0.0161 (18)
C50	0.0207 (12)	0.0236 (15)	0.0258 (13)	0.0028 (11)	0.0003 (10)	0.0029 (11)
Co1	0.0269 (3)	0.0223 (3)	0.0237 (3)	0.0004 (2)	0.00182 (19)	0.0014 (2)
N1	0.0252 (12)	0.0271 (13)	0.0307 (12)	0.0007 (9)	0.0033 (9)	0.0002 (9)
N2	0.0315 (14)	0.0433 (16)	0.0461 (16)	0.0071 (12)	−0.0069 (12)	−0.0035 (13)
O1	0.0242 (9)	0.0268 (10)	0.0264 (10)	−0.0013 (8)	0.0093 (8)	0.0018 (8)
O2	0.0491 (13)	0.0258 (12)	0.0478 (13)	−0.0078 (10)	0.0238 (10)	−0.0055 (9)
O3	0.0347 (12)	0.0444 (14)	0.0466 (13)	0.0077 (11)	−0.0200 (10)	−0.0051 (11)
O4	0.0511 (15)	0.0463 (14)	0.0473 (14)	0.0075 (11)	−0.0141 (11)	−0.0127 (12)
O5	0.0390 (11)	0.0199 (11)	0.0365 (12)	0.0029 (9)	0.0150 (9)	0.0008 (9)

### Geometric parameters (Å, °)

**Table:**

C1—N1	1.338 (3)	C25—H25A	0.9700
C1—C4	1.360 (4)	C25—H25B	0.9700
C1—H1	0.9300	C26—C27	1.373 (4)
C2—C3	1.373 (4)	C26—C31	1.382 (4)
C2—C4	1.389 (4)	C27—C28	1.362 (4)
C2—C6	1.498 (4)	C27—H27	0.9300
C3—C5	1.352 (4)	C28—C29	1.361 (4)
C3—H3A	0.9300	C28—H28	0.9300
C4—H4	0.9300	C29—C30	1.378 (5)
C5—N1	1.335 (4)	C29—C32	1.483 (4)
C5—H5A	0.9300	C30—C31	1.383 (4)
C6—C7	1.472 (5)	C30—H30	0.9300
C6—H6	0.9300	C31—H31	0.9300
C7—C8	1.520 (4)	C32—O4	1.209 (4)
C7—H7	0.9300	C32—O3	1.290 (4)
C8—C12	1.367 (5)	C50—O2	1.249 (3)
C8—C9	1.367 (5)	C50—O1	1.262 (3)
C9—C10	1.394 (4)	Co1—O5	2.092 (2)
C9—H9	0.9300	Co1—O5^i^	2.092 (2)
C10—N2	1.324 (4)	Co1—O1^i^	2.1149 (17)
C10—H10	0.9300	Co1—O1	2.1149 (17)
C11—N2	1.303 (4)	Co1—N1^i^	2.141 (2)
C11—C12	1.378 (4)	Co1—N1	2.141 (2)
C11—H11	0.9300	O3—H3	0.8200
C12—H12	0.9300	O5—H5C	0.73 (4)
C25—C26	1.514 (4)	O5—H5D	0.98 (4)
C25—C50	1.522 (4)		
			
N1—C1—C4	124.8 (3)	C28—C27—C26	121.5 (3)
N1—C1—H1	117.6	C28—C27—H27	119.2
C4—C1—H1	117.6	C26—C27—H27	119.2
C3—C2—C4	116.3 (3)	C29—C28—C27	121.4 (3)
C3—C2—C6	118.6 (3)	C29—C28—H28	119.3
C4—C2—C6	125.0 (3)	C27—C28—H28	119.3
C5—C3—C2	120.8 (3)	C28—C29—C30	118.7 (3)
C5—C3—H3A	119.6	C28—C29—C32	120.5 (3)
C2—C3—H3A	119.6	C30—C29—C32	120.8 (3)
C1—C4—C2	119.1 (3)	C29—C30—C31	119.5 (3)
C1—C4—H4	120.5	C29—C30—H30	120.3
C2—C4—H4	120.5	C31—C30—H30	120.3
N1—C5—C3	124.0 (3)	C26—C31—C30	121.8 (3)
N1—C5—H5A	118.0	C26—C31—H31	119.1
C3—C5—H5A	118.0	C30—C31—H31	119.1
C7—C6—C2	117.1 (3)	O4—C32—O3	122.1 (3)
C7—C6—H6	121.4	O4—C32—C29	123.0 (3)
C2—C6—H6	121.4	O3—C32—C29	114.7 (3)
C6—C7—C8	115.2 (3)	O2—C50—O1	125.6 (2)
C6—C7—H7	122.4	O2—C50—C25	118.2 (2)
C8—C7—H7	122.4	O1—C50—C25	116.1 (2)
C12—C8—C9	117.1 (3)	O5—Co1—O5^i^	180.00 (10)
C12—C8—C7	124.0 (3)	O5—Co1—O1^i^	92.48 (8)
C9—C8—C7	118.9 (3)	O5^i^—Co1—O1^i^	87.52 (8)
C8—C9—C10	119.4 (3)	O5—Co1—O1	87.52 (8)
C8—C9—H9	120.3	O5^i^—Co1—O1	92.48 (8)
C10—C9—H9	120.3	O1^i^—Co1—O1	180.00 (7)
N2—C10—C9	122.9 (3)	O5—Co1—N1^i^	93.70 (9)
N2—C10—H10	118.5	O5^i^—Co1—N1^i^	86.30 (9)
C9—C10—H10	118.5	O1^i^—Co1—N1^i^	89.61 (8)
N2—C11—C12	124.1 (3)	O1—Co1—N1^i^	90.39 (8)
N2—C11—H11	117.9	O5—Co1—N1	86.30 (9)
C12—C11—H11	117.9	O5^i^—Co1—N1	93.70 (9)
C8—C12—C11	119.6 (3)	O1^i^—Co1—N1	90.39 (8)
C8—C12—H12	120.2	O1—Co1—N1	89.61 (8)
C11—C12—H12	120.2	N1^i^—Co1—N1	180.00 (13)
C26—C25—C50	110.9 (2)	C5—N1—C1	115.0 (2)
C26—C25—H25A	109.5	C5—N1—Co1	122.57 (19)
C50—C25—H25A	109.5	C1—N1—Co1	122.36 (19)
C26—C25—H25B	109.5	C11—N2—C10	116.8 (3)
C50—C25—H25B	109.5	C50—O1—Co1	127.08 (17)
H25A—C25—H25B	108.1	C32—O3—H3	109.5
C27—C26—C31	117.0 (3)	Co1—O5—H5C	138 (3)
C27—C26—C25	122.4 (3)	Co1—O5—H5D	100 (2)
C31—C26—C25	120.6 (3)	H5C—O5—H5D	107 (3)

Symmetry codes: (i) −*x*, −*y*+1, −*z*.

### Hydrogen-bond geometry (Å, °)

**Table:**

*D*—H···*A*	*D*—H	H···*A*	*D*···*A*	*D*—H···*A*
O5—H5C···O1^ii^	0.73 (4)	2.13 (4)	2.822 (3)	158 (4)
O5—H5D···O2^i^	0.98 (4)	1.74 (4)	2.610 (3)	145 (3)
O3—H3···N2^iii^	0.82	1.85	2.667 (3)	173.

Symmetry codes: (ii) −*x*, −*y*+2, −*z*; (i) −*x*, −*y*+1, −*z*; (iii) −*x*+1, *y*+1/2, −*z*+1/2.
